# Eating attitudes and behaviors characteristics in high-level Kenyan female and male distance runners

**DOI:** 10.3389/fspor.2026.1756302

**Published:** 2026-04-01

**Authors:** Lauri Õnnik, Martin Mooses, Silva Suvi, Diresibachew W. Haile, Robert Ojiambo, Amy R. Lane, A. C. Hackney

**Affiliations:** 1 University of Tartu, Tartu, Estonia; 2 Moi University, Eldoret, Kenya; 3 Addis Ababa University, Addis Ababa, Ethiopia; 4 University of Global Health Equity, Butaro, Rwanda; 5 University of North Carolina, Chapel Hill, NC, United States

**Keywords:** dietary practices, elite runners, health, high-level athletes, REDs

## Abstract

**Background:**

East African middle- and long-distance runners have dominated international running events for decades. This success is likely derived from the combination of genetics, training, environment, lifestyle, and social factors. Physiological capacity and training regimes-histories have been reported extensively, however, research addressing lifestyle factors is relatively scant. Examination of eating behaviors and attitudes is based upon small sample sizes, and nutritional behaviors are compared and framed relative to European or Western country cohorts.

**Methods:**

Therefore, we chose to conduct surveys of select aspects of eating attitudes and behaviors with a focus on DE/ED (disordered eating/eating disorder) risks in a group of high-level Kenyan female and male distance runners (Athletes; *n* = 50) in comparison to healthy, but Sedentary (*n* = 58), Kenyan females/males. Participants visited our laboratory areas (Eldoret or Nairobi) and were provided with an eating attitudes and behaviors questionnaire (EAT-26) to complete.

**Results:**

Within the three EAT-26 sub-scores, only the Dieting and Oral Control components displayed significant differences. Specifically, the female Sedentary group had higher scores (*p* < 0.01) than the female Athletes for Dieting; while the male Athlete group had higher scores for Oral Control (*p* < 0.05) than the male Sedentary group and also there was a trend in male Athletes group to have a higher proportion of individuals with ≥20 points on the EAT-26 (i.e., diagnostic cut-point for increased risk of DE/ED.

**Conclusions:**

A greater Oral Control component sub-score is associated with “self-control of eating and the perceived pressure from others to gain weight.” The very slightly elevated sub-score observed in this score and the trend of a greater 20-point proportion do suggest, to a small degree, that our male Athletes might be at an increased risk of DE/ED behaviors. However, female Athletes displayed lower risk than their respective Sedentary counterparts.

## Introduction

East African middle- and long-distance runners, especially those from Kenya and Ethiopia, are well known as they have dominated international running events for decades (1). For example, Kenyan runners (male and female) have collectively won over 60 Olympic medals in middle and long-distance running since the 1996 Olympic Games in Atlanta. Billat et al. postulated such success is likely derived from the combination of genetics, training, environment, lifestyle, and social factors (2). Specific elements to these points; e.g., physiological capacity and training regimes-histories, have been reported extensively in the literature (1, 3–5). To a lesser extent, however, the research addressing lifestyle factors is relatively scant.

All endurance athletes are greatly affected by their nutritional choices and behaviors, and it is well established that poor nutritional practices can lead to reduced performance as well as health consequences as outlined in the recent 2023 IOC Consensus Statement on Relative Energy Deficiency in Sports (REDs) publication (6). Relative to East African runners there are several reports available about aspects of their energy expenditure as well as dietary composition (i.e., macro- and micro-nutrients) (5, 7–9). However, examination of eating behaviors and attitudes in these cohorts is based upon small sample sizes. Nonetheless, such perspectives are critical as they provide insight as to whether such athletes are at risk for unhealthy situations [e.g., the development of disordered eating (DE), and potential manifestations of a clinical diagnosis of eating disorders (ED)] as well as whether their dietary habits are driven by intentions (i.e., “Theory of Planned Behaviour”) or other factors (10). Both DE and ED are of critical importance as they are risk factors for REDs as well as the Female Athlete Triad (FAT), which, as noted, are associated with compromised athlete well-being (6). Interestingly, in many such published reports on African runners, their nutritional behaviors are compared and framed relative to European or Western country cohorts upon which many nutritional guidelines (especially in relation to athletes and sports) are established, which creates possible complications due to societal and cultural differences between comparison groups.

In light of the above, we chose to conduct surveys of select aspects of eating attitudes and behaviors with a focus on DE/ED risks in a group of high-level Kenyan female and male distance runners in comparison to healthy, but sedentary, Kenyan females/males.

## Methods

### Participants and study design

The runner participants in the study were recruited from their training camps in the Eldoret area, Kenya, while the sedentary participants were from the Eldoret and Nairobi areas of Kenya. All participants received written and oral information about the study before signing an informed consent statement. They were allowed to withdraw from the study at any time. Our research design approach was that of a cross-sectional observational study (i.e., analyzing data from a population at a single point in time to measure the prevalence of health outcomes and describe features of that population).

The runner participants were middle- and long-distance athletes who were of high-level competitive backgrounds (competitors at the national and international level). All runners were required to be actively engaged in training and competing at the time of the study for middle- to long-distance athletics events, had done so for several years, and were viewed by their coaches as high-level performers (i.e., national and international levels).

The seasonal best running performance of each participant was assessed using the International Association of Athletics Federations (IAAF) Scoring Tables (11). These scoring tables assign a numerical score to each performance, enabling comparison between events and participants. The sedentary participants did not engage in any regular physical activity other than ambulatory aspects of their daily lives (e.g., walking to a bus stop), and were not former competitive athletes. Participants in each group (runners, sedentary) were healthy and reported no major clinical medical problems during screening, nor experienced any musculoskeletal injuries at the time of the study. English language proficiency was necessary for inclusion in the study.

For the Athlete group 50 data records were accepted for analysis (22 female and 28 male), while in the Sedentary group, 58 data records were accepted (25 female and 33 male). The mean IAAF scores equate to the following performance times: males = 13:30 5 km, 28:27 10 km and 2:13:31 marathon; females = 16:15 km, 34:17 km, and 2:41:40 marathon. These performance times place our athlete groups at the Tier 4 “Elite/International Level,” and Tier 5 “World Class” according to the classification system of McKay et al. (12).

Participants visited our laboratory areas (Eldoret or Nairobi) and were provided with an eating attitudes and behaviors questionnaire (EAT-26) to complete (including name, age, and sex). During these visits, members of the research team were available to address any queries about the questionnaire items as the participants completed them. Participants were allowed as much time as necessary to complete questionnaires. Furthermore, native language speakers were also available in case any translation issues arose. The questionnaire completed by the participants is overviewed in the following section. Additionally, at these visits, the height and weight of the participants were assessed [methods reported previously by Õnnik et al. (13)] to calculate their body mass index (BMI). Blood samples were obtained from a vein in the antecubital region after an overnight fast and before the exercise testing. Haematological measurements were performed on whole blood, and hormonal measurements were performed on plasma specimens.

### Eating attitudes test (EAT-26) questionnaire

The EAT-26 questionnaire is a self-report measure comprised of attitudinal items and behavioral frequency items designed to identify the presence of DE (e.g., guilt about eating, dieting, avoidance of foods) and ED (e.g., anorexia nervosa, bulimia nervosa, and binge eating disorder) risks (13). Specifically, there are 26 self-report questions assessing general eating. The total score provides an overall risk score, with higher scores indicating a greater risk of an DE/ED (see following section).

### EAT-26 score definitions

The psychometric properties of the EAT-26 have been evaluated, and the instrument has been validated against other instruments (14–17). This holds for both the general population and athletes (18). It has been used in many studies investigating the DE/ED risk in athletes. All 26 items allow the response categories: “never”, “rarely”, “sometimes”, “often”, “usually” and “always”. The first three are scored as 0 and the following as 1, 2, and 3, respectively. Recommendations indicate that participants with a score of 20 or above should be further investigated for diagnostic criteria of EDs (17, 19). Scores between 10 and 19 points might be suggestive of DE (20). Additionally, EAT-26 responses generate three sub-scores reflecting the constructs of 1) “dieting”, 2) “bulimia and food preoccupation” and 3) “oral control”. According to Garner et al., the first construct relates to “an avoidance of fattening foods and a preoccupation with being thinner”, the second covers “items reflecting thoughts about food as well as those indicating bulimia”, and the third relates to “self-control of eating and the perceived pressure from others to gain weight” (14).

Per literature recommendations, the results of the EAT-26 were interpreted with caution as they represent screening tools rather than diagnostic criteria. Consequently, in the context of this study, the EAT-26 is used as a standardized self-reported questionnaire and the values are interpreted as measurements of the individual ED/DE risk (21).

At the time of this study, all participants were eating a “free-living”, self-selected diet representative of their usual daily approaches to eating. They were instructed not to consume or attempt any special dietary practices. Select aspects of food consumption practices have been published elsewhere (13).

### Maximal oxygen uptake testing

To assess the fitness level of the athlete groups, as there is a high association between the incremental laboratory exercise tests and competition performances (22), participants were subjected to a running-based maximal oxygen uptake test (VO_2max_). They reported to our laboratory in a rested, fasted state. Following the warm-up period (10 min), participants performed an incremental running test on a motorized treadmill (Cardionics Type 3113, Sweden) until voluntary exhaustion. Before starting the test, each participant remained stationary on the treadmill for 3 min and resting cardio-respiratory measures were collected. The initial running speed was set at 8 km·h^−1^ for females and 10 km·h^−1^ for male participants at a gradient of 1% (23). The running speed was increased 2 km·h^−1^ every 3 min until 16 km·h^−1^ for females and 18 km·h^−1^ for male participants. After completing the 3 min stage at 16 km·h^−1^ for female and 18 km·h^−1^ for male participants, respectively, the speed remained constant until the end of the test; however, the elevation was increased 1% after each minute until voluntary exhaustion was reached (24). Heart rate and expired gases were measured continuously using MetaMax 3B (Cortex Biophysic GmbH, Leipzig, Germany), which was calibrated before each test according to instructions by the manufacturer. The highest average oxygen uptake (VO_2_) during a 30 s period as well as a failure to further increase VO_2_ consumption despite an increase in the work rate was defined as the VO_2max_ (25).

### Statistical analysis

All questionnaires were scanned for completeness and accurate recording of answers. In cases of errors or incomplete data, participants were contacted about correcting their responses, but if they declined to do so, their questionnaire was removed from the data set.

Total scores, the three sub-set scores for the EAT-26 were calculated for each participant in the Athlete and Sedentary subject groups. Furthermore, the frequency of total scores at or exceeding 20 was determined for each group by sex. Means and standard deviations were calculated from all these obtained values. Group response comparisons for statistical significance were performed within sexes only using independent sample t-tests, Mann–Whitney *U*-Tests, Chi-square, and Odds Ratio (OR) tests where appropriate for specific data. Additionally, where appropriate, confidence intervals (CI, 95%) and effect sizes (Hedge's *g*) were calculated. Statistical significance was set at *p* ≤ 0.05.

## Results

The physical characteristics of the participants appear in Table 1. In the male groups, the Athletes were significantly older and had lower body weight and BMI values than the Sedentary (*p* ≤ 0.05). In the female groups, the Athletes had lower body weight and BMI values than the Sedentary (*p* ≤ 0.05). The effect sizes for these differences ranged from small to large. The mean years in training, IAAF scores, and the VO_2max_ obtained from treadmill testing appear in Table 1 (no between-sex comparison was conducted). Table 2 presents select hormone, haematological, and bone mineral density values for both female and male athlete and sedentary groups.

**Table 1:** Physical characteristics of all participants, as well as select physiological/training characteristics of athletes, and the EAT-26 score responses of all participants.

Measurement	Males			Female		
	Athlete (*n* = 28)	Sedentary (*n* = 33)	Mean difference, CI, *g*	Athlete (*n* = 22)	Sedentary (*n* = 25)	Mean difference, CI, *g*
Age (y)	28.00 ± 3.77*	23.75 ± 3.71	−4.25 −6.17, −2.33 1.12	28.18 ± 6.64	25.56 ± 5.97	−2.62 −6.32, 1.08 0.41
Height (cm)	171.67 ± 5.12	168.84 ± 7.91	−2.83 −6.31, 0.65 0.41	163.63 ± 6.29	165.76 ± 6.17	2.13 −1.54, 5.80 −0.05
Weight (kg)	57.50 ± 6.72	62.54 ± 9.52*	5.04 0.74, 9.34 −0.60	51.63 ± 4.02	63.47 ± 9.76*	11.84 7.34, 16.33 −1.52
BMI (kg/m^2^)	19.50 ± 1.98	22.13 ± 4.76*	2.63 0.70, 4.56 −0.69	19.29 ± 1.37	23.10 ± 3.34*	3.81 2.27, 5.35 −1.43
Training (y)	7.86 ± 3.85	NA		7.93 ± 7.97	NA	
IAAF Score	1100.3 ± 60.7	NA		1021.0 ± 128.4	NA	
VO_2max_ (mL/kg/min)	67.0 ± 4.5	NA		52.1 ± 5.8	NA	
EAT-26 Total Score	17.92 ± 10.64	13.39 ± 7.48	−4.53 −9.19, 0.13 0.49	12.22 ± 9.34	19.20 ± 9.27*	6.98 1.50, 12.46 −0.74
EAT-26 Scores (≥20)	10/28 (35.7%)	5/33 (15.2%)		5/22 (22.7%)	10/25 (40.0%)	
EAT-26 Sub-score (*Dieting*)	9.89 ± 6.27	8.12 ± 5.36	−1.77 −4.74, 1.21 0.30	6.95 ± 5.63	12.96 ± 6.16**	6.01 2.52, 9.49 −1.00
EAT-26 Sub-score (B*ulimia & food preoccupation*)	3.38 ± 2.82	2.24 ± 2.43	−1.14 −2.48, 0.20 0.43	2.40 ± 3.45	2.52 ± 2.23	0.12 −1.57, 1.81 −0.04
EAT-26 Sub-score (*Oral control*)	4.75 ± 3.55*	3.03 ± 2.27	−1.72 −3.22, −0.22 0.58	2.86 ± 3.85	3.72 ± 3.34	0.86 −1.25, 2.97 −0.24

CI, 95% confidence interval; *g*, Hedge's effect size*; NA, non-applicable.

* *p* ≤ 0.05.

** *p* ≤ 0.01.

*Effect size magnitude (*g*): small = 0.2, medium = 0.5, large = 0.8 [see reference (35)].

**Table 2:** Select hormone, haematological, and bone mineral density values for the athlete and sedentary (control) groups used in the study (not all subjects completed all measures). These data have been reported previously and are presented here only for informational purposes. For further details on analysis and interpretation, see reference (13).

Measure	Male ♂		Female ♀		Probability (A vs. S within sex)
	Athlete (A)	Sedentary (S)	Athlete (A)	Sedentary (S)	
Hormone					
LH (U/L)	5.23 ± 2.29	4.62 ± 1.66	7.50 ± 14.25	8.86 ± 13.84	0.248 ♂ 0.733 ♀
FSH (U/L)	2.67 ± 1.16	2.23 ± 1.0	10.4 29.4	8.2 ± 18.2	0.134 ♂ 0.749 ♀
Testosterone (nmol/L)	25.25 ± 6.91	25.13 ± 5.96	0.78 ± 0.95	0.78 ± 0.81	0.944 ♂ 0.977 ♀
Haematological					
Haemoglobin (g/dL)	16.3 ± 1.0	16.7 ± 1.6	14.0 ± 1.7	14.0 ± 2.1	0.339 ♂ 0.951 ♀
Haematocrit (%)	47.6 ± 2.7	49.8 ± 3.9	42.1 ± 4.3	43.5 ± 7.0	0.014 ♂ 0.540 ♀
Total body bone mineral density (*Z*-score frequency distribution)*					
*Z* > −1	29	26	23	25	0.293 ♂ 0.999 ♀
*Z* −1 to −2	1	3	3	3	
*Z* < −2	0	0	0	1	

LH, luteinizing hormone; FSH, follicle-stimulating hormone.

* A bone *Z*-score of −2.0 or below is considered “poor” for bone density, indicating a significantly lower bone mass than expected for someone of comparable age.

### EAT-26 responses

Table 1 also reports the overall total EAT-26 scores, the frequency of scores at or above 20 points as well as the three sub-set scorings associated with the questionnaire.

Relative to the total scores, no significant differences were found between the male groups. However, in the females, the Sedentary group had significantly higher total scores than the Athletes (*p* < 0.05; effect size magnitude approached large). For the frequency of the scores above 20 points, no significant differences were found within the male or female Athlete groups vs. the Sedentary groups. There was a trend for a prevalence (%) of 20-point (or greater) scores to be more frequent in the male Athlete group (*p* = 0.07). Subsequent analysis of this data revealed the OR between Athlete and Sedentary groups were non-significant; males = 2.36 (CI: 0.72, 7.71; *p* = 0.16), and females = 0.56 (CI: 0.17, 1.90; *p* = 0.36).

Within the three EAT-26 sub-score categories, only the Dieting and Oral Control components displayed significant differences (effect size magnitude, medium to large). Specifically, the female Sedentary group had higher scores (*p* < 0.01) than the female Athletes for Dieting; while the male Athlete group had higher scores for Oral Control (*p* < 0.05) than the male Sedentary group.

## Discussion

The high success rate of female and male Kenyan distance runners at the international level has made these athletes known and noted worldwide. Success in distance running can primarily be distilled down essentially to being a function of genetics, training, environment, and lifestyle choices (e.g., dietary practices, sleep) (26). Many of these elements of Kenyan athletes have been studied, but aspects of their dietary attitudes, especially relative to their native country women/men are lacking. As such, we chose to survey eating attitudes and behaviors using the EAT-26 question in such athletes compared to healthy, but sedentary Kenyan counterparts. From our survey, perhaps the most interesting finding was the lack of substantial indicators of DE or ED risks in our female or male athlete groups; although the existence was not null. Conversely, in our female sedentary participants, such risks were displayed, although on a limited basis.

Prior research on eating attitudes and behaviors in Kenyan runners has tended to focus on female athletes. This has occurred due to the perceived risk of DE or ED development being greater in females and the likelihood of menstrual dysfunction and osteopenia development associated with DE/ED (27). To that extent, Muia et al. used the *Eating Disorder Inventory* subscales and the *Three-Factor Eating Questionnaire's* cognitive dietary restraint subscale to assess the DE status of adolescent Kenyan female runners (the authors denoted as “elite”) in comparison to non-athletes (28). Levels of subclinical and clinical DE were similar between athletes and non-athletes (no significant differences). This finding does not agree with ours *per se* but does support our outcomes that the athletes were not at a greater risk for eating behavior abnormalities than their non-athlete counterparts. Similarly, Hulley et al. found elite female Kenyan distance runners had the least eating disorder psychopathology traits compared to similar athletes from the United Kingdom (“elite” as defined by the authors) (29). These authors concluded that participation in distance running at an elite level does not in itself predispose athletes to a DE/ED. All the parameters we evaluated from our EAT-26 assessment would also support this claim for our female athletes. Why our Kenyan Sedentary group showed more psychopathology traits towards eating is not entirely clear. However, some reports indicate that Western body ideals, specifically thinness, have had strong influences on the African women population and as such, potentially swayed their attitudes/behaviors toward eating and food in general (30, 31).

We were somewhat surprised by our finding of the male Athlete group having a significantly higher level on the third sub-score (oral control) and a tendency (N.B., albeit *p* > 0.05 < 0.10) in this group to have a higher proportion of individuals with ≥20 points on the EAT-26. As noted, this oral control sub-score has been related to “self-control of eating and the perceived pressure from others to gain weight” (14). The very slightly elevated sub-score and the trend of ≥20-point proportionality might suggest, to a small degree, our male athletes might be at an increased risk of DE/ED behaviors. However, due to the limitations within our study data (see subsequent section), we cannot be definitive in the magnitude of this risk level, but this is an interesting finding. That said, we advise such athletes and their medical care team to be mindful of the health consequences of DE/ED development and be vigilant to take necessary steps if warranted. If not, the development of both short- and long-term health consequences in athletes can manifest. In particular, for example, modern evidence supports that dietary practices (i.e., attitudes and behaviors) can compromise sporting performance, through the development of the Overtraining Syndrome, FAT, or REDs. Each of the latter is associated with endocrinological disruptions, poor bone health, and major reproductive dysfunctions (32, 33). To that end, we did report in a prior publication that the athlete and sedentary individuals (female and male) in this study were found to have no major acute/chronic abnormalities (i.e., not reaching diagnostic clinical criteria) in the parameters just noted (i.e., comparison of respective values between athletes and sedentary controls were highly similar) (13). To support this point, Table 2 presents select data on bone mineral content, hematological, as well as hormonal measures between the groups [methodological procedures reported elsewhere by Õnnik et al. (13)].

As with any study, there are both strengths and limitations to our research. It is highly valuable and informative that our athlete samples are of a high-performance level relative to training and competitive backgrounds. That is, access to such select athletes can be difficult for researchers (34). Furthermore, gaining access to them in their home country's location allowed reflection and responses of real-life scenarios and not while away competing internationally, where lifestyles/behaviors can be very atypical. Furthermore, the utilization of Kenyan country persons as a comparison (i.e., control) group allowed for a more appropriate culturally and societal-based assessment (although we acknowledge our cultural validity is limited and needs to be addressed in future work). Conversely, though, we acknowledge the sample size recruited was smaller than desired, especially relative to female athletes, a group that has been studied far too little. Furthermore, our participants could, for the most part, be viewed as a sample of convenience, presenting potential “sampling bias”. While the EAT-26 is a well-validated screening instrument, it is, however, not diagnostic, and certain subscales, like Oral Control, may reflect performance-related dietary restraint (driven by intentions—a distinct possibility in the current data) rather than disordered eating in elite endurance athletes, which limits the interpretation of our findings beyond prevalence reporting. Furthermore, sex specific findings should be interpreted cautiously, as group differences in age, body composition, and training characteristics (i.e., potential data confounders), especially among male athletes, may influence EAT-26 responses and reflect performance-related norms rather than eating pathology. Finally, since our survey questionnaire was a self-report, we must accept the possibility that “response bias” may have influenced our data.

## Conclusions

In conclusion, ample evidence supports that endurance athletes, such as runners, are at risk for developing DE/ED. This is especially true for female athletes. The development of DE/ED in athletes can compromise sporting performance and overall health and well-being. The current data herein on Kenyan runners suggest an extremely mild risk of DE/ED may exist for males, but that females actually display less risk than their respective sedentary counterparts (N.B., although no major health abnormalities were noted between groups). However, caution is advised in the interpretation of this data due to the limitations of the study design and protocol; therefore, as such, future investigations pursuing this topic are still warranted.

## Data Availability

The raw data supporting the conclusions of this article may be requested from the authors.
